# Cardiovascular Effects of Salvianolic Acid B

**DOI:** 10.1155/2013/247948

**Published:** 2013-06-10

**Authors:** Jie Wang, Xingjiang Xiong, Bo Feng

**Affiliations:** Department of Cardiology, Guang'anmen Hospital, China Academy of Chinese Medical Sciences, Xicheng District, Beijing 100053, China

## Abstract

Salvianolic acid B (SAB, Sal B) is the representative component of phenolic acids derived from the dried root and rhizome of *Salvia miltiorrhiza* Bge (Labiatae) which has been used widely and successfully in Asian countries for clinical therapy of various vascular disturbance-related diseases for hundreds of years. However, its exact cardioprotective components and the underlying mechanism for therapeutic basis are still poorly understood. This paper discussed and elucidated the underlying biological mechanisms and pharmacology of Sal B and their potential cardioprotective effects.

## 1. Introduction

**Figure 1:**
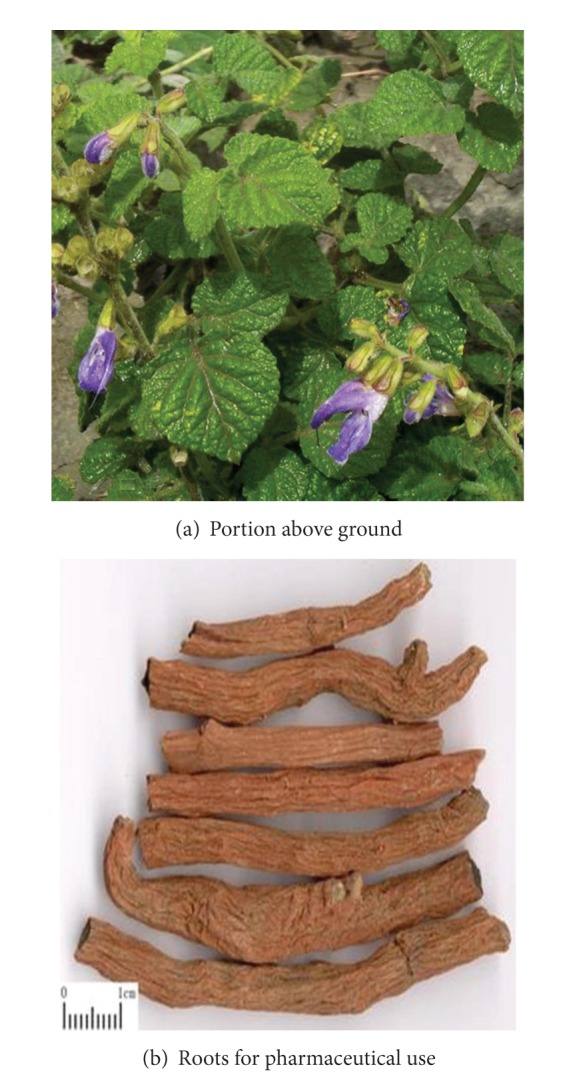
Morphology of Salvia miltiorrhiza.

Salvia is the Salvia miltiorrhiza's dried roots and rhizomes which belongs to plants of Labiatae *Lagurus* grass species (see Figure 1). The herb is some bitter and slightly cold in flavor and enters heart and liver meridian. Salvia is a commonly used herbal medicine for “invigorating” the blood and reducing blood clotting in eastern countries, particularly in China. Currently, it is widely used for the treatment of cardiovascular diseases (CVDs) and cerebrovascular diseases [1–3] and is gaining more and more popularity both in eastern and western countries, including the United States, European countries, and so forth. Furthermore, it can exert protecting effect on liver [4–6], kidneys [7–9], and lungs [10, 11], especially improving ischemia- /reperfusion- (I-/R-) induced injury. According to the theory of traditional Chinese medicine (TCM), it has the effect of promoting blood circulation to clear blood stasis, regualting menstruation and relieving analgesia, clearing heart fire and calming the nerves. Modern pharmacology studies have shown that Salvia has many pharmacological effects, such as increasing coronary blood flow, reducing excitability and conductivity of myocardial, protecting against myocardial ischemic/reperfusion injury, improving microcirculation, antiplatelet aggregation and thrombosis, protecting and improving the kidney function, and reducing blood viscosity as well as antibacterial, anti-inflammatory and antioxidant protection against brain tissue I/R injury.

**Figure 2:**
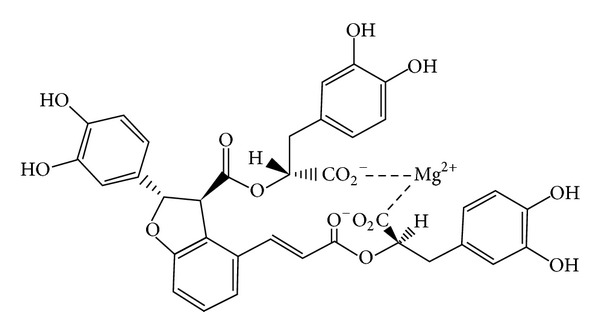
Salvianolic acid B-Major water-soluble compounds derived of Salvia miltiorrhiza.

Salvia has been used for various diseases related to blood stasis syndrome in China for thousands of years, and now, it is widely used for CVDs [12]. During the past 60 years, much significant progress has been made from theory, experiments to clinic fields based on the inherit, and innovation of thoughts in TCM to clarify the treatment principle and method of Salvia and Salvia preparations, which has already got consensus and increasing popularity in medical community in China. Currently, a growing number of medical researchers have focused on the chemical constituents of Salvia. Main chemical constituents of Salvia miltiorrhiza roots extract are classified into two major categories: water-soluble compounds (WSC) and lipophilic diterpenoid quinines (LDQ) [13]. According to the pharmacological structure of phenolic acid compounds, we can further divide the WSC and LDQ into single phenolic acids (protocatechuic aldehyde, protocatechuic acid, caffeic acid, and 3,4-dihydroxyphenyl lactic acid) and polyphenolic acids (rosmarinic acid, lithospermic acid, salvianolic acid A, salvianolic acid B, and other salvianolic acids). The major LDQs are tanshinone I (TsI), tanshinone IIA (TsIIA), tanshinone IIB (TsIIB), and other tanshinones [14]. In recent years, studies both in vivo and in vitro have confirmed that salvianolic acid can regulate the signal transduction pathways of vascular endothelial cells, vascular smooth muscle cells, and cardiac cells to prevent and treat cardiovascular damage [15]. Currently, preparations derived from Salvia are widely used in clinical treatment with symptoms or diagnosis of coronary heart disease, chest tightness, and angina embolism. The most commonly used formulations of Salvia are injections, dipping pills, and so on, including, Danshen injection, the Salvia infusion injection, the salvianolate injection (lyophilized), Xiang Dan injection, Danxiang Guanxin injection, Danhong injection (infusion injection), and Danshen dripping pill (DSP). Currently, with increasing studies on Salvia miltiorrhiza including randomized controlled trials (RCTs) and systematic reviews (SRs), DSP which is composed of Salvia miltiorrhiza (Danshen) is apparently more effective than ISDN (isosorbide dinitrate) in treating angina pectoris [16]. As we know, the main constituents of Salvia are water-soluble components, such as 3,4-dihydroxyphenyl lactic acid (also called danshensu), salvianolic acid A, salvianolic acid B, and so on. Salvianolic acid B is the main constituent of Salvia phenolic acid and the most active constituent of water-soluble salvianolic acid substances. Salvia phenolic acids could elevate the ability of antioxidation, affect the blood lipid metabolism, and inhibit the generation atherosclerosis, which basically represents the traditional role of the activating blood circulation and dissolving stasis of Salvia in TCM [17]. Salvianolic acid B, also known as satanic acid B or lithospermic acid B, is a condensate of three molecules danshennol and one molecule of caffeic acid (see Figure 2). It is a pale yellow amorphous powder in character. In this paper, the pharmacology of salvianolic acid B in the treatment for CVDs was reviewed.

## 2. Cardiovascular Pharmacology

### 2.1. Antioxidant Effect

 The oxidative stress of organism can produce a large number of reactive oxygen species (ROS), which can lead to ischemic cardiomyopathy through direct or indirect way. So the antioxidative stress is an important part of protecting ischemic myocardium. Salvianolic acid B is a new generation of the natural antioxidants, which typically presents in the form of a metal salt, especially, magnesium salts. This compound has a plurality of phenolic hydroxyl group, so it has strong antioxidant activity. There are at least six experiments demonstrate the antioxidant role of salvianolic acid B. It can influence Ca^2+^ aggregation and endothelial cell NO release of hypoxia/reoxygenation-induced cell. When acid B concentration is 2.5, 5, and 10 mg/l, cell viability and superoxide dismutase (SOD) activity are enhanced, and the formation of malondialdehyde (MDA) in human umbilical vein endothelial cells (ECV304) is inhibited. Hypoxia/reoxygenation stimulation can increase the expression of human umbilical vein endothelial intracellular Ca^2+^ concentration, NO release, and eNOS mRNA, but reduce the expression of iNOS mRNA. SalB can alleviate damage of the hypoxia/reoxygenation stimulation to ECV304 cell, increase the release of NO which is closely related to alleviation of cell damage [18]. SalB inhibits HG-induced oxidative stress and reduces the generation of ROS and 8-hydroxy-2-deoxyguanosine (8-OHDG) and mitochondrial depolarization and apoptosis in a dose-dependent manner. It can downregulate the expression of Bax and AIF nuclear translocation and cytochrome c release mediated by HG, but upregulate the expression of Bcl-2 induced by HG. Besides, SalB attenuated HG-induced caspase of the enzyme 3, 9 and minimize PARP cleavage of Schwann cells (SCs). SalB antagonist oxidative stress, mitochondrial activation pathway, and apoptosis of SCs are induced by high glucose [19]. SalB inhibits angiotensin II or H_2_O_2_ and TNF-*α*-induced gelatinolytic activity in human aortic smooth muscle cells (HASMCs) in a concentration-dependent manner because salvianolic acid B scavenged H_2_O_2_ in a dose-dependent manner in test tube [20]. One research showed that both SalB and EGb 761 were able to scavenge O_2_^+^ and OH, inhibit lipid peroxidation of microsomes, and protect SH-SY5Y cells against H_2_O_2_-induced oxidative damage. SalB exerts more antioxidant efficiency than EGb 761 [21]. Both salvianolic acid B and danshensu exhibit higher scavenging activities against free hydroxyl radicals (HO), superoxide anion radicals (O^2−^), 1,1-diphenyl-2-picryl-hydrazyl (DPPH) radicals, and 2-azino-bis (3-ethylbenzthiazoline-6-sulfonic acid) (ABTS) radicals and weaker iron chelating and hydrogen peroxide (H_2_O_2_) scavenging activities than vitamin C [22]. Antioxidant effect of salvianolic acid B in vitro is shown in Table 1.

### 2.2. Antiplatelet Aggregation, Anticoagulant, and Antithrombotic Effect

Platelet plays a key role in platelet thrombosis. Many thrombotic diseases have hyperthyroidism characteristics of aggregation of platelet releasing, so inhibiting of platelet aggregation is of great significance for the prevention of CVDs. Previous studies have shown that salvianolic acid B can inhibit platelet aggregation and adhesion, and the progression is related to integrin *α*2*β*1, but the specific mechanism of action is still unclear. Salvianolic acid B controlled more than 20 kinds of protein expression, such as 70 kDa heat shock protein, forest domain protein CLP36 copine I, peroxiredoxin-2, coronin-1 B, and cytoplasmic dynein intermediate chain 2C. The experiments predict and verify that integrin *α*2*β*1 may be the target of salvianolic acid B. Integrin protein signaling cascade network includes regulation intracellular levels of Ca^2+^ and cytoskeleton-related proteins such as coronin-1B and cytoskeleton structure of platelets [23]. SalB inhibits platelet aggregation and activation in patients and stabilizes plaque by reducing MMP-9 and improve prognosis [24]. Other studies about salvianolic acid B on eNOS activity of platelet endothelial cell platelet concluded that certain dose concentration range (<10 mg/l) of salvianolate can significantly increase eNOS activity and promote the production of L-citrulline and the release of NO, which can inhibit Ca^2+^ transmembrane transport of platelet to inhibit platelet aggregation and thrombosis [25–27]. SAB and tetramethylpyrazine (TMP) could inhibit shear-induced platelet aggregation (SIPA) with a dose-dependent manner in SD rats. Magnesium lithospermate B (MLB) inhibits the aggregation and 5-HT release in rabbit platelets and attenuates intracellular calcium concentration by inhibiting the rise of [Ca^2+^]_*i*_ in thrombin stimulated platelets but decreases the [Ca^2+^]_*i*_ in resting platelets [28]. SAB inhibited static platelet adhesion to a synthetic peptide specific for the collagen receptor *α*2*β*1 and binding of an antibody against *α*2*β*1 to platelets and inhibited the interaction of soluble *α*2*β*1 to immobilized collagen in a solid phase [29]. Antiplatelet aggregation, anticoagulant, and antithrombotic effect of salvianolic acid B in vitro and in vivo is shown in Table 2.

### 2.3. Promoting Cardiac Angiogenesis

Coronary revascularization surgery has resolved the problem of epicardial vascular occlusion, but no-reflow, reperfusion injury, restenosis, stent thrombosis, and other clinical tricky problems still remained as a pressing issue. The pathomechanism is directly related to the formation of collateral circulation and coronary microcirculation and endothelial cell injury. Therefore, it is of particularly importance to promote formation of collateral circulation and angiogenesis in myocardial ischemic area currently. Salvianolic acid B and Danshen crude extract can promote cell growth and differentiation. SalB can upregulate matrix metalloproteinase 2 (MMP-2) gene and upregulate vascular endothelial growth factor (VEGF) and vascular endothelial growth factor receptor R2 (VEGF-R2) genes [30]. Promoting cardiac angiogenesis effect of salvianolic acid B in vitro is shown in Table 3.

### 2.4. Protecting Myocardial Cells from Apoptosis

Apoptosis is an important mechanism of acute myocardial ischemia and reperfusion myocardial cell death. Excessive accumulation of ROS leads to oxidative stress. The progression can induce cell death process after regulating series of Intracellular signaling pathways [31], such as PI3 K/Akt pathway, TAB1-P38 apoptosis signaling pathway, and caspase-3 apoptotic pathway. In these pathways, the mitogen-activated protein kinases (MAPKs) and phosphatidylinositol-13-kinase (PI3 K)/Akt pathway play a major role in cell growth, survival, differentiation, and apoptosis [32]. Studies showed that PI3 K inhibitor (LY294002) prevents ERK pathway activation induced by hydrogen peroxide and protects cells from apoptosis, and SalB could inhibit H_2_O_2_-induced cell apoptosis mainly through the PI3 K/Akt pathway (ERK upstream) [33]. Salvianolic acid B can significantly reduce the myocardial infarct size and blood lactate dehydrogenase level of model rat with acute myocardial infarction. Further studies showed that SalB can enhance cell activity and reduce the number of sub-G1 and apoptotic nuclei of ischemic cell model in order to show its antiapoptotic effects. The specific mechanism is as follows: salvianolic acid B specifically inhibits phosphorylation of p38 mediated by TAB1 (TGF-*β*-activated protein kinase 1 binding protein 1) by interfering with the interaction of TAB1 and P38 [34]. One research showed that the concentration of acid B is higher in the acute myocardial infarction rats model compared with nonischemic myocardial area, indicating that salvianolic acid B can improve cardiac function and myocardial tissue structure. Biochemical analysis showed salvianolic acid B can regulate the expression of 36 kinds of proteins in rats with AMI, which is composed of the mesh part of the diagram of cell's apoptosis and metabolism. Salvianolic acid B can also inhibit polymerase 1 pathway, improve the integrity of the mitochondria and nuclei in heart tissue of acute myocardial infarction, and protect myocardial cells from apoptosis [35]. The experiment proved that treatment of 50 uM LAB can significantly reduce death. LAB significantly reduced phosphorylation of p38 and JNK induced by cytokine, which is in accordance with *β*-cells decrease in cleaved caspase-3 activity by a significant activation expression of of Nrf2-HO-1 (the heme oxygenase 1) and SIRT-1. LAB also has a protective effect on cytokine-induced caspase-3 apoptotic pathway [31]. Hunger for three hours can lead to myocardial cells induced autophagy, which is an important reason for myocardial cell's damage. Salvianolic acid B can protect of starving cells and inhibit of apoptosis process by blocking early stages of autophagic flux, respectively [36]. One research showed PC12 cells pretreated with SalB (10 nmol/L, 100 nmol/L, 1 mol/L) manifested relatively low proportion of apoptosis (15.7%, 13.5%, 11.8%). The mechanism is that Par-4 is involved in the protective effect of SalB against A-beta-induced damage while salB can largely prevent the increase in Par-4 expression of the A-beta-induced PC12 cells. Magnesium lithospermate B exhibits direct superoxide radicals scavenging and xanthine oxidase inhibitory activity [37]. The conclusion can be verified by experiments of protecting HL-60 cells from superoxide radicals-induced apoptosis in the xanthine oxidase reaction [38]. SM treatment is able to induce the highest frequency of apoptosis in cholesterol-fed balloon-injury rabbits, upregulate the expression of p53 and the frequency of TUNEL-positive cells [39]. SMND-309, is a new derivate of salvianolic acid B. It can prevent the elevation in ST segment level and the increase in serum creatine kinase-MB, lactate dehydrogenase, alanine aminotransferase and cardiac troponin T content, increase the activities of superoxide dismutase, catalase and glutathione peroxidase, decrease the content of malondialdehyde in myocardium, reduce the myocardium necrosis scores and the number of apoptosis cardiocytes, upregulated the expression of antiapoptotic protein, Bcl-2; and downregulate the expression of proapoptotic protein, Bax [40]. Protecting myocardial cells from apoptosis of salvianolic acid B in vivo and in vitro is shown in Table 4.

### 2.5. Inhibiting Ischemia and Hypoxia of Myocardial Injury

Myocardial ischemia and hypoxia diseases such as coronary heart disease threaten human health severely. Both physicians and researchers have made great effort in looking for effective drug of anti-ischemic hypoxic/hypoxia. Salvianolic acid B could antagonize voltage-dependent Ca^2+^ channels and therefore synergistically reduce cardiac ischemic injury with the antioxidant effects [41]. Other researches also studied the protective effect of salvianolic acid B on NO. The research confirmed for the first time that salvianolic acid B and tanshinone IIA promote left-handed arginine (L-arginine) uptake by enhancing expression of catalase (CAT) and increasing phosphorylation of eNOS through AMPK-PI3 K-Akt signaling pathway. Results showed that NO is a key factor for salvianolic acid B to reverse myocardial ischemia and hypoxia damage [42]. In the early stages of LPS-induced neonatal rat cardiomyocytes injury, TLR4-NF*κ*B-TNF*α* signaling pathway which is not directly related to this process with HSP70 is activated quickly. Mechanism of salvianolic acid B protection of the ischemic myocardium is related to suppressing TLR4-NF*κ*B-TNF*α* signaling pathway in dose-dependent manner [43]. SalB exerts cardioprotective effect on large MI mediated by reversing upregulation of leptin, endothelin pathways and oxidative stress, and recovering the normal expressions of SERCA2a and PLB in myocardium [44]. MLB may protect the heart from ischemic/reperfused injury by decreasing apoptosis through the inhibition activity of JNK3 [45]. Magnesium lithospermate B can induce eNOS expression in the endothelial cells of BAs and improve endothelial dysfunction. MLB inhibits ET-1 production in SAH animals via an NO-dependent mechanism [46]. Inhibiting ischemia and hypoxia of myocardial injury of salvianolic acid B in vitro is shown in Table 5.

### 2.6. Endothelial Cell Protection

Under normal circumstances, vascular endothelial secretion of vasoactive substances, which regulate vasomotor to protect the vessel wall from the infiltration of inflammatory cells, could inhibit thrombosis and vascular smooth proliferation of muscle cell. Many factors can cause vascular endothelial injury and dysfunction. It is the the first stage of atherosclerosis. The endothelial cell protection role of salvianolic acid B is essential for the occurrence and development of atherosclerosis. Studies revealed that when the concentration of SME is 50 mg/mL and 100 mg/mL and concentrations of salvianolic acid B were 1, 2.5, 5, 10, 20 mg/mL, the expression of the VCAM-1 was lower, and the expression of ICAM-1 was also significantly reduced in a dose-dependent manner. SME may exert endothelial cell protection role by downregulating VCAM-1 and ICAM-1 in dose-dependent manner [47]. Salvianolic acid B can reduce the endothelial dependent vasodilation decline of Otsuka Long-Evans Tokushima Fatty (OLETF) rat, but increase the level of serum nitrite and lower serum AGEs concentration. The mechanism is related to Akt phosphorylation as well as reducing the O bit N-acetylglucosamine amine of eNOS. The mechanism is also related to increasing expression of 3-phosphoinositide kinase/Akt signaling pathway-dependent Nrf-2 as well as reducing the oxidative stress caused by hyperglycemia and apoptosis of vascular endothelial cell [48]. Salvianolic acid B exerts protective effect on vascular endothelial cells by inhibiting TNF-*α*-induced PAI-1 (plasminogen activator inhibitor type 1) mRNA production and protein secretion [49]. SalB induces the expression of GRP78 by activating ATF6 and the PERK-eIF2a-ATF4 pathway and protects human endothelial cells from oxidative stress-induced cellular damage [50]. Endothelial cell protection of salvianolic acid B in vivo and in vitro is shown in Table 6.

### 2.7. Improving Hemorheology

Pharmacological studies have shown that the change of blood flow state is one of the important causes of thrombosis. And fibrinogen plays an important role in platelet aggregation, and so reducing fibrinogen can reduce thrombosis in a certain sense. Salvianolic acid B and paeonol compounds can significantly decrease the fibrinogen and malondialdehyde levels in a dose-dependent manner, increase high-density lipoprotein levels, improve the rabbit blood viscosity and plasma viscosity, decrease NO/ET proportion, and decrease lactate dehydrogenase (LDH) and creatine phosphokinase (CPK) levels in a dose-dependent manner. It is proved that salvianolic acid B can improve blood hemorheology, reduce oxidative damage, improve the vascular endothelial cell function, and prevent the development of coronary artery disease [51]. SalB increased the fibrinolytic and anticoagulant potential of cultured HUVECs by upregulating the expression of t-PA and TM and by downregulating the expression of PAI-1 [52]. Both DLA and SAB can inhibit venular thrombosis induced by photochemical reaction (PR) thrombosis in rat mesentery and delay thrombus-initiation time [53]. Improving hemorheology of salvianolic acid B in vivo is shown in Table 7.

### 2.8. Acting on Ion Channel Function

Many ion channels are closely related to cardiovascular disease. It is unclear whether and how MLB affects the cardiac ion channels. The occurrence of cardiovascular disease may be limited to not only a single ion channel, but to multiorganization, multicell network level of ion channel interactions. Recently, many researchers focus on ion channels and regulatory proteins associated. Whether the ion channel leads to cardiovascular disease by causing arrhythmogenic is not yet formed as a conclusion. There are at least two experiments about SalB acting on the BK_Ca_ channel. One experiment confirmed MLB can make arterial vasodilating through the activation of BK_Ca_ channel (big-conductance Ca^2+^-activated K^+^ channels) of smooth muscle cell and the increase of endothelial NO release [54]. Another one verified that salvianolic acid B could activate the opening of the BK_Ca_ channels of the porcine coronary artery smooth muscle cells. Cumulative application of salvianolic acid B (30–300 *μ*M) caused an L-NNA- (100 *μ*M) insensitive potentiation of the outward BK_Ca_ (iberiotoxin-sensitive Ca^2+^-activated K^+^) current amplitude. Salvianolic acid B (300 *μ*M) caused an ODQ-sensitive enhancement of the outward BK_Ca_ current amplitude [55]. An experiment stimulated SH-SY5Y neuroblastoma cells in tumor cells with different concentrations of ouabain or MLB, using Fluo4-AM (fluorescent dye) measurements to measure Ca^+^ level of cells. It is confirmed that elevation of ouabain and MLB can cause increase of intracellular Ca^2+^ levels, which may be related to inhibition activity of Na^+^/K^+^-ATPase enzyme [56]. MLB reversibly inhibited L-type Ca^2+^ current (*I*_Ca,L_) on single ventricular myocytes of adult guinea pigs. The inhibition was use dependent and voltage dependent and the voltage-dependent Ca^2+^ antagonistic effect of MLB works in concert with its antioxidant action for attenuating heart ischemic injury. When the concentration of MLB is up to 300 AM, there is no significant effect on the fast-inactivating Na^+^ current (*I*_Na_), but on delaying rectifier K^+^ current (*I*_K_) and inward rectifier K^+^ current [57]. The vasorelaxant effects of salvianolic acid B were produced by inhibition of Ca^2+^ influx in the vascular smooth muscle cells. The opening of K^+^ channels had a minor contribution to their effects [58]. Acting on ion channel function of salvianolic acid B in vitro is shown in Table 8. 

### 2.9. Anti-Inflammatory Effect

Various researches demonstrated that inflammatory response was involved in the process of myocardial infarction (MI), endothelium injury, atherosclerosis, and cardiovascular hypertrophy [59, 60], which have been mostly introduced in the former paragraphs. Adhesion and migration of white blood cells in the vessel wall is an early manifestation of atherosclerosis formation. The use of antioxidants to inhibit the expression of adhesion molecules can prolong the progression of atherosclerosis. Salvianolic acid B is considered to be promising powerful antioxidants. One research study mechanism of salvianolic acid B and Salvia hydroalcoholic extract (SME) to TNF-*α* induced HAECs. When concentration of salvianolic acid B is 0.48 times, it can significantly inhibit nuclear factor *κ*B (NF-*κ*B) activity of TNF-*α*-induced HAECS. It confirmed the exact anti-inflammatory effect of salvianolic acid B [61]. Shih Chung Chen have proved that salvianolic acid B significantly inhibits the phosphorylation of JAK2 (tyrosine 1007/1008) and STAT1 (Tyr701 and serine 727 (Ser727)) induced by IFN-*γ*. The specific mechanism may be that salvianolic acid B inhibits STAT1 downstream target chemoattractant factor IP-10, MIG, I-TAC induced by IFN-*γ* and inhibits the secretion of promoter activity of IP-10 and IP-10 protein. Salvianolic acid B can also reduce the adhesion role of monocyte to endothelial cells when endothelial cells stimulated with IFN-*γ* were used as experimental object. Salvianolic acid B also increases PIAS1 and SOCS1 expression. This may contribute to its inhibition of JAK-STAT1 signaling pathway [62]. SalB significantly reduced the production of NO, TNF-*α*, IL-1b, and ROS induced by LPS treatment in rat primary microglia in a dose-dependent manner [63]. The activation of T lymphocytes contributes to the inflammatory processes of atherosclerotic diseases. MLB inhibits IL-2, IL-4, TNF-*α*, and interferon-gamma production; reduces the expressions of T cell activation markers CD 25 and CD 69; downregulates activator protein-1 (AP-1), nuclear factor kappa B (NF-*κ*B), and octamer binding transcription factor (Oct-1) DNA-binding activity, and also inhibits c-Jun N-terminal kinase (JNK), I*κ*B*α* degradation, nuclear translocation of p65 and p50, and decreased I*κ*B*α* kinase (IKK) activity through suppressing JNK-AP-1, IKK-I*κ*B*α*–NF-*κ*B, and Oct-1 signaling pathways [64]. SalB suppresses the expression of proinflammatory cytokines TNF-*α*, IL-1, and enhance and the expression of anti-inflammatory cytokines IL-10 and TGF-*β*1. All of these findings extended the protective role of SalB in the model of TBI [65]. SalB treatment also suppressed the pathway of ERK1/2, JNK, and p38 mitogen-activated protein kinase. It can also attenuate the increase in prostaglandin E2 production and NADPH oxidase activity in LPS-treated HASMCs [66]. SalB and LSS treatment inhibit TNF-*α*-induced NF-*κ*B activation evidenced by I*κ*B*α* degradation and p65 nuclear translocation in HAECs. SalB has a combination effect with LSS to reduce the expression of three adhesion molecules (VCAM-1, ICAM-1, and E-selectin), leading to reduced monocyte adhesion to HAECs [67]. Anti-inflammatory protection role of Salvianolic acid B in vitro is shown in Table 9.

### 2.10. Preventing Cell Migration, Proliferation, and Intimal Hyperplasia

Proliferation of vascular smooth muscle cells (VSMC) and migration of platelet-derived growth factor (PDGF) play an important role in the development of atherosclerosis and restenosis. One in vitro research studied the therapeutic potential of neointimal formation of salvianolic acid B to carotid artery injury rat and the PDGF signaling pathway which stimulates the proliferation of vascular smooth muscle cell and migration. It is demonstrated that SalB directly scavenges reactive oxygen species in the system of xanthine oxidase and reduces the generation of reactive oxygen species in the PDGF-BB-induced vascular smooth muscle cells. In rat carotid artery balloon-injury model, SalB plays an important role in preventing the formation process of neointimal mediated by injury and prevents proliferation and migration of vascular smooth muscle cell in vitro mediated by PDGF-BB. In view of this, it is believe that salvianolic acid B has prospects in the prevention of atherosclerosis and postangioplasty restenosis [68]. SDF-1*α* significantly promotes growth and migration of A10 cells, while SalB can significantly reverse the impact of costimulation group. Similarly, SalB significantly downregulated the upregulation Raf-1, MEK, and ERK1/2 phosphorylation of ERK1/2, FAK, and phosphorylated FAK stimulated by CXCR4 SDF-1*α* and increased activity of NF-*κ*B promoter. In addition, SalB is also effective in reducing intimal hyperplasia induced by balloon angioplasty. In short, SalB can prevent cell proliferation, migration, and subsequent neointimal hyperplasia. This pharmacological mechanism can be explained by theory of inhibiting receptor expression levels of the CXCR4 and expression of downstream molecularSDF-1*α*/CXCR4 [69]. SalB could inhibit high glucose-induced human mesangial cells proliferation and extracellular matrix production in a dose-dependent manner through modulating the cell-cycle progress and MMP-2 and MMP-9 activities via suppressing NF-*κ*B activation [70]. Preventing cell migration, proliferation, and intimal hyperplasia salvianolic acid B in vitro is shown in Table 10.

### 2.11. Antiatherosclerosis

Atherosclerosis is characterized by the lipoid calming on affected artery intima, complex carbohydrates accumulateing, and middle arterial disease. Coronary atherosclerosis is of great harm, which could lead to the stenosis or obstruction of blood vessels. Currently, it is demonstrated that SalB can act on Nrf2-ARE signaling pathway and p38-MAPK signaling pathway to prevent the occurrence of atherosclerotic disease. SalB can also activate NAD(P)H quinine oxidoreductase-1 (NQO1) by pathway of nuclear factor erythroid 2-related factor-2 antioxidant responsive element (Nrf2-ARE), thereby inhibiting the vascular injury and vascular smooth muscle cell proliferation and migration. It might be the potential molecular target of salvianolic acid B against atherosclerosis [71]. Anti-atherosclerotic of salvianolic acid B also has relation with inhibition of H-monDC mature. The oxidation of low-density lipoprotein (ox-LDL) can promote the mature of H-monDC, stimulate cells expression of CD40, CD86, CD1a, HLA-DR and IL-12, IL-10, production of TNF-*α* and upregulate signaling pathway. SalB can suppress the above process and activate PPAR*γ* nuclear translocation in order to reduce the ox-LDL-induced upregulation of TLR4 and primary reactive protein 88 myeloid differentiation, and also inhibit downstream p38-MAPK signaling cascade pathway [72]. Salvianolic acid B can antagonize lipid uptake process of Scavenger receptor mediated by CD36 and reduce low density lipoprotein (mLDL) uptake in a dose-dependent manner in phorbol-12-myristate-13-acetate (PMA)-stimulated THP-1 and RAW 264.7 cells, thus preventing of atherosclerotic disease [73]. SalB significantly attenuate upregulations of both MMPs and the LPS-induced cell migration as well as downregulation of the extracellular-signal-regulated kinase1/2 (ERK1/2) and c-Jun NH2-terminal kinase (JNK) [74]. Antiatherosclerosis of Salvianolic acid B in vitro is shown in Table 11.

### 2.12. Inhibiting Left Ventricular Remodeling

Acute myocardial infarction may lead to left ventricular remodeling, and then cause congestive heart failure. Therefore, it is necessary to study treatment strategies of inhibiting left ventricular remodeling. Salvianolic acid B could selectively inhibit the activity of MMP-9 in a rat model of myocardial infarction. Salvianolic acid B can also effectively increase the thickness of the left ventricular wall in the myocardial infarction rats to improve the contraction of the heart, and reduce cardiac fibrosis. Previous experiments confirmed the exact role of anti-cell fibrosis of salvianolic acid B, but the specific mechanism of action was still unclear. There are a variety of hypotheses [75]. Salvianolic acid B inhibits the synthesis of type I collagen of non-TGF-*β*1 stimulated human hepatic stellate cell line (LX-2), the anti-fiber of mechanism is related to direct inhibiting p38 signaling pathway and cross effect of the Smad to ERK signaling pathway. Cardiac fibroblasts play a key role in cardiac function. As we all know, MMP-9 greatly influence the occurrence and development of cardiac remodeling [76]. One study about the catalytic MMP-9 CD (domain of MMP-9) and neonatal cardiac fibroblasts showed 200 nm MMP-9 CD can stimulate cardiac fibroblast migration; increase collagen synthesis; upregulate secretion of ICAM, TNF-*α*, IL-6 and VCAM-1; and downregulate the expression of VEGF. This is closely related to cell proliferation [77]. SalB can inhibit A-beta aggregation and fibril formation and the cellular toxicity of aged A-beta towards PC12 cells [78]. Antimyocardia fibrosis, inhibiting left ventricular remodeling of salvianolic acid B in vivo and in vitro is shown in Table 12.

### 2.13. Antiarrhythmic

It is generally believed that the anti-arrhythmic and local anesthetics drugs mainly act on voltage-gated NA^+^ channels. A new view showed NA^+^ channel agonist has the positive inotropic effect. Salvianolic acid B is regarded as a new kind of NA^+^ channel agonist. It can slow down the inactivation of NA^+^ channel and increase action potential duration (APD). One research proved dmLSB has no apparent influence to currents of K^+^ channels or Ca^+^ channel; it only selectively affects NA^+^ current (*I*_NA_). dmLSB slows down *I*_NA_ kinetics inactivation by increasing the proportion of material that cannot cause persistent sodium electricity loss of live. dmLSB only prolongs APD and then affects EAD. It is different from other NA^+^ channel agonists which impact EAD and cause arrhythmia. Therefore, the clinical use of dmLSB is more safer and more promising [79]. Salvianolic acid B has the same molecular mechanism of inhibition of Na^+^-K^+^-ATP enzyme activity with cardiac glycosides. And MLB has lower cytotoxic effect than ouabain, so it will become great potential substitutes for cardiac glycosides with a wide range of clinical trials. Anti-arrhythmic effect of salvianolic acid B in vitro is shown in Table 13.

## 3. Discussion and Perspective

Currently, the high incidence of cardiovascular diseases (CVDs) worldwide potentially threaten human health [80–84]. The prevalence of CVDs is incessantly increasing and it is still the most common cause of death. History of application of herbal medicine as representative of complementary and alternative medicines in China has been lasting for thousands of years. Traditional Chinese medicine (TCM) has also formed a particular way which other therapeutics cannot match with it on diagnosis and treatment of the disease. And a variety of practices including Chinese herb and formulas, acupuncture, moxibustion, cupping, qigong, Tai Chi, diet, and exercise therapy were originated in China [85–87]. Nowadays, Chinese scholars combine traditional Chinese medicine with modern medicine perfectly carrying forward integrative mode. It takes the advantage of theory and practice of Chinese and modern medicine and exerts dual effect to improve clinical therapy efficacy. Chinese scholars have make great achievement on reducing the mortality and improving the quality of life by using patterns of integrative mode on diagnosis and treatment of cardiovascular, cerebrovascular disease and the tumor disease. And study on the blood stasis syndrome (BSS) and promoting blood circulation and removing blood stasis (PBCRBS) is the most active field of research of integration of traditional and western medicine in China [88, 89]. Scholars studying herbs of accelerating blood circulation (ABC) have made remarkable achievements in recent years [90, 91].

Many Chinese herbal medicine have function of accelerating blood circulation, clearing blood stasis, and dredging the meridians, such as Danshen, chuanxiong, chishao, and honghua. Herbal medicines are great treasure that nature gifts to human and have made great contribution to human health [92–95]. Conclusive evidence can be found in the prevention and treatment of cardiovascular disease whether from traditional medicine or modern pharmacology research perspective. Salvia is the most widely used traditional Chinese medicine in the field of cardiovascular and cerebrovascular diseases. Currently, with increasing popularity of complementary and alternative medicine among CVDs patients, constituents of Chinese herb formulas are the key research areas [96, 97]. Many researches demonstrated that Chinese herbs can definitely regulate whole body by acting on multilevel and multitargets. Among them, salvianolic acid B is a water-soluble antioxidant from Salvia extract. It plays significant role of antioxidant effect; antiplatelet aggregation, anticoagulant, and antithrombotic effect; promoting cardiac angiogenesis; antiatherosclerosis; protecting myocardial cells from apoptosis; inhibiting left ventricular remodeling; inhibiting ischemia and hypoxia of myocardial injur; and protection of endothelial cell. SalB also has the protection effect of anti-arrhythmic, improving hemorheology; acting on ion channel function anti-inflammatory protection, and preventing cell migration, proliferation, and intimal hyperplasia. Though SalB has so many effects on preventing and treatment of CVDs, there are also some problems we need to arise to develop both efficacious and pharmaceutical medicines. On current, Research about role of anti-inflammatory and effect of protecting myocardial cells from apoptosis were performed more frequently than other studies. Nevertheless there is only a few studies published about the promoting cardiac angiogenesis and anti-arrhythmic effect. Also there is deficiency of in vivo research on effect of antioxidant; anti-arrhythmic; antiatherosclerosis, promoting cardiac angiogenesis and preventing cell migration, proliferation, and intimal hyperplasia. So, further systematic in vivo researches are warranted to explore and verify the potential effect to provide precise guidance for clinical use and new drug discovery. Furthermore, there is also no randomized controlled trials (RCTs) and systematic reviews (SRs) about SalB. So, it is imperative to conduct multicentered, large-sized samples and randomized and arid controlled trials to reasonably evaluate the efficacy and safety of Chinese herb and formulas for CVDs. As we know, active ingredients with potential protecting and treating CVDs are material basis of Chinese herb and formulas [98, 99]. However there are so many active ingredients in Chinese herb, so large quantity of active ingredients should be identified, extracted; and purified. Correspondingly, more research should be designed and complemented to explain the mechanism of each agent. All the above problems seriously limit the research and progress on CVDs treatment and should be solved as soon as possible in future researches.

## Figures and Tables

**Table 1:** Antioxidant effect of salvianolic acid B in vitro.

Cells/tissues	Effects	Reference
ECV304	Activity of SOD, release of NO, aggregation of Ca^2+^.	Luo et al., 2002 [18]

SCs	Generation of ROS, generation of 8-OHDG, expression of Bax, AIF nuclear translocation, cytochrome C release, caspase of the enzyme 3, 9, cleavage of PARP, expression of Bcl-2.	Sun et al., 2012 [19]

HASMCs	Activity of gelatinolytic, scavenge H_2_O_2_.	Zhang and Wang, 2006 [20]

SH-SY5Y	Scavenge O_2_^+^ and OH, reduce oxidative damage.	Liu et al., 2006 [21]

Note: HEK293T cell: human embryonic kidney cells; HO-1: heme oxygenase-1; ROS: reactive oxygen species; Nrf2: nuclear factor 2–related factor 2; SOD: superoxide dismutase; MDA: malondialdehyde; ECV304: human umbilical vein endothelial cells; TMP: tetramethylpyrazine; HASMCs: human aortic smooth muscle cells; 8-OHDG: 8-hydroxy-2-deoxyguanosine; SCs: Schwann cells; MMP-2: matrix metalloproteinase-2; PARP: poly ADP-ribose polymerase.

**Table 2:** Anti-platelet aggregation, anticoagulant, and antithrombotic effect of salvianolic acid B in vitro and in vivo.

Type	Cells/tissues	Effects	Reference
In vitro research	Human platelet endothelial cell	Activity of eNOS, L-arginine to L-citrulline, release of NO.	Radomski et al., 1990 [25, 26]
	Platelet	Collagen receptor *α*2*β*1.	Wu et al., 2008 [29]

Type	Organ/animals	Effects	Reference

In vivo research	Rats blood Rats blood	Induce SIPA. 20 kinds of protein expression, *α*2*β*1 integrin protein, levels of intracellular Ca^2+^, cytoskeleton-related proteins, cytoskeleton platelets structure.	Li et al., 2004 [28] Ma et al., 2011 [23]

Note: eNOS: endothelial nitric oxide synthase; L-arginine: left-handed arginine; SIPA: shear-induced platelet aggregation.

**Table 3:** Promoting cardiac angiogenesis effect of salvianolic acid B in vitro.

Cells/tissues	Effects	Reference
HUVEs	Expression of MMP-2 gene, expression of VEGF, VEGF gene, VEGF receptor 2 genes.	Lay et al., 2003 [30]

Note: VEGF: vascular endothelial growth factor receptor; MMP-2: matrix metalloproteinase-2; HUVEC: human umbilical vein endothelial cells.

**Table 4:** Protecting myocardial cells from apoptosis of salvianolic acid B in vivo and in vitro.

Type	Cells/tissues	Effects	Reference
In vitro research	rCMECs	PI3K/Akt pathway, ERK upstream.	Blanc et al., 2003 [32]
	H9C2	TAB1-P38 apoptosis signaling pathway, cell activity, reduce sub-G1, inhibit p38 phosphorylation, interaction of TAB1 and P38.	Du et al., 2010 [34]
	INS-1	Phosphorylation of p38, phosphorylation of JNK, expression of Nrf2-HO-1, expression of SIRT-1, caspase-3 apoptotic pathway.	Han et al., 2011 [36]
	PC12	Expression of Par-4, superoxide radicals scavenging, xanthine oxidase inhibitory activity.	Tang and Zhang, 2002 [37]
	HL-60	Superoxide radicals-induced apoptosis, xanthine/xanthine oxidase reactions.	Liu et al., 2009 [38]
	Rabbit neointimal cell	Expression of p53, frequency of TUNEL-positive cells.	Hung et al., 2001 [39]

Type	Organ/animals	Effects	Reference

In vivo research	Rat heart	Myocardial infarct size, blood lactate dehydrogenase.	Du et al., 2010 [34]
	Rat heart	Cardiac function, myocardial tissue structure, expression of 36 kinds of proteins, ADP-ribose polymerase-1 pathway, integrity of mitochondria, integrity of nuclei.	Xu et al., 2011 [35]
	Rat heart	ST segment level, serum creatine kinase-MB, lactate dehydrogenase, alanine aminotransferase, cardiac troponin T content, activities of superoxide dismutase, activities of catalase, activities of glutathione peroxidase, expression of anti-apoptotic protein, expression of Bcl-2, expression of proapoptotic protein, expression of Bax.	Yang et al., 2010 [40]

Note: Nrf2: nuclear factor 2-related factor 2; TAB1: TGF-*β*-activated protein kinase 1 binding protein 1; rCMECs: rat cerebral microvascular endothe-lial cells; APD: action potential duration; PI3K: phosphatidylinositol-13-kinase; ERK: extracellular-signal-regulated kinase.

**Table 5:** Inhibiting ischemia and hypoxia of myocardial injury of salvianolic acid B in vitro.

Type	Cells/tissues	Effects	Reference
In vivo research	Guinea pig heart	Anti-voltage-dependent Ca^2+^ channels.	Wang et al., 2006 [41]
	Rat heart	Upregulation of leptin, upregulation of oxidative stress, endothelin pathways, expressions of SERCA2a, expressions of PLB.	He et al., 2008 [44]
	SAH rat	Induce eNOS expression, improve endothelial dysfunction, ET-1 production.	Chang et al., 2011 [46]

Type	Organ/animals	Effects	Reference

In vitro research	HUVEs	Promote L-arginine uptake, expression of catalase (CAT), phosphorylation of eNOS, AMPK-PI3K-Akt signaling pathway, NO.	Pan et al., 2011 [42]
	Neonatal rat cardiomyocytes	TLR4-NF*κ*B-TNF*α* signaling pathway.	Wang et al., 2011 [43]

Note: SAH: subarachnoid hemorrhage; TNF-*α*: tumor necrosis factor *α*; HUVEC: shuman umbilical vein endothelial cells; NF-*κ*B: nuclear factor *κ*B; TLR: toll-like receptor; eNOS: endothelial nitric oxide synthase.

**Table 6:** Endothelial cell protection of salvianolic acid B in vivo and in vitro.

Type	Cells/tissues	Effects	Reference
In vitro research	HAECs	Expression of VCAM-1, expression of ICAM-1,	Chen et al., 2001 [47]
	HUVECs and HAECs	Akt phosphorylation, O bit N-acetylglucosamine amine of eNOS, 3-phosphoinositide kinase/Akt signaling pathway, expression of Nrf-2	Kim et al., 2010 [48]
	HUVECs	Expression of PAI-1 Mrna.	Zhou et al., 2005 [49]
	HUVECs	Expression of GRP78, ATF6, and the PERK-eIF2a-ATF4 pathway.	Wu et al., 2009 [50]

Type	Organ/animals	Effects	Reference

In vivo research	OLETF Diabetic rat	Endothelial dependent vasodilation, levels of serum nitrite, serum AGEs concentration, reduce the oxidative stress.	Kim et al., 2010 [48]

Note: HUVEC: human umbilical vein endothelial cells; HAEC: human aortic endothelial cells; OLETF: Otsuka Long-Evans Tokushima Fatty; PAI-1: plasminogen activator inhibitor type 1; eNOS: endothelial nitric oxide synthase; VCAM-1: vascular cell adhesion molecule-1; ICAM-1: intercellular adhesion molecule 1.

**Table 7:** Improving hemorheology of salvianolic acid B in vitro and in vivo.

Type	Cells/tissues	Effects	Reference
In vitro research	HUVECs	Expression of t-PA and TM, expression of PAI-1.	Wang et al., 2009 [53]

Type	Organ/animals	Effects	Reference

In vivo research	Rabbits heart	Decrease fibrinogen and level, decrease malondialdehyde level, increase high-density lipoprotein level, improve rabbit blood viscosity, improve rabbit plasma viscosity, decrease NO/ET proportion, decrease LDH, decrease CPK.	Shi et al., 2007 [52]

Note: CPK: creatine phosphokinase; LDH: lactate dehydrogenase.

**Table 8:** Acting on ion channel function of salvianolic acid B in vitro.

Type	Cells/tissues	Effects	Reference
In vitro Research	SH-SY5Y	Suppress Na^+^-K^+^-ATP enzyme, increase intracellular Ca^2+^ levels.	Chen et al., 2010 [56]
	Porcine CASMs	Activate BK_Ca_ channels, L-NNA insensitive, BK_Ca_ current amplitude, inhibited L-type Ca^2+^ current, delay rectifier *I*_K_, inward rectifier *I*_K_.	Lam et al., 2006 [55]
	VSMCs	Activation of BK_Ca_ channel, increase NO release.	Zhang et al., 2010 [54]

Type	Organ/animals	Effects	Reference

In vivo research	Rat coronary artery	Inhibition of Ca^2+^ influx.	Lam et al., 2006 [58]

Note: VSMC: vascular smooth muscle cells; BK_Ca_: iberiotoxin-sensitive Ca^2+^-activated K^+^ current; *I*_Ca,L_: L-type Ca^2+^ current; *I*_Na_: Na^+^ current; *I*_K_: K^+^ current.

**Table 9:** Anti-inflammatory effect of salvianolic acid B in vitro.

Type	Cells/tissues	Effects	Reference
In vitro research	HAECs	Activity of NF-*κ*B.	Sun et al., 2011 [61]
	Endothelial cells	JAK-STAT1 signaling pathway, inhibit phosphorylation of JAK2, inhibit phosphorylation of STAT1, inhibit IP-10, MIG, and I-TAC, reduce adhesion role of monocyte to endothelial cells, increase expression of PIAS1, increase expression of SOCS1.	Chen et al., 2006 [66]
	Rat primary microglia	Production of IL-1b and ROS, production of NO, TNF-a, inhibit IL-2, IL-4, TNF-*α*.	Cheng et al., 2012 [64]
	Human peripheral T lymphocyte	Expressions of CD 25 and CD 69, down-regulate AP-1, NF-*Κ*b, down-regulate Oct-1DNA-binding activity, inhibit c-JNK, I*κ*B*α* degradation, inhibit nuclear translocation of p65 and p50, IKK activity, suppress JNK-AP-1, IKK-I*κ*B*α*-NF-*κ*B and Oct-1 signaling pathways, phosphorylation of ERK1/2.	Chen et al., 2011 [65]
	HASMCs	Phosphorylation of JNK, pathway of ERK1/2, c-JNK, and p38 MAPK, prostaglandin E2 production, NADPH oxidase activity.	Chen et al., 2006 [66]
	HAECs	NF-*κ*B activation, VCAM-1, ICAM-1, and E-selectin.	Xie et al., 2010 [67]

Type	Organ/animals	Effects	Reference

In vivo research	Mice brain	Expression of TNF-*α*, expression of IL-1, expression of IL-10 and TGF-*β*1.	Chen et al., 2006 [66]

Note: PAI-1: plasminogen activator inhibitor type 1; NF-*κ*B: nuclear factor *κ*B; HAEC: human aortic endothelial cells; p38 MAPK: p38 mitogen-activated protein kinase.

**Table 10:** Preventing cell migration, proliferation, and intimal hyperplasia salvianolic acid B in vitro.

Cells/tissues	Effects	Reference
rVSMCs	PDGF signaling pathway, scavenge ROS, system of xanthine oxidase, generation of ROS, process of neointimal, prevent proliferation, prevent migration.	Hur et al., 2008 [68]

A10	Expression of SDF-1*α*/CXCR4, regulate Raf-1 and MEK, regulate ERK1/2 and phosphorylation ERK1/2, regulate FAK and phosphorylated FAK, activity of NF-*κ*B promoter.	Pan et al., 2012 [69]

Human mesangial cells	Modulate the cell-cycle progress, activity of MMP-2, activity of MMP-9, suppress NF-*κ*B activation.	Luo et al., 2008 [70]

Note: ROS: reactive oxygen species; MMP-2: matrix metalloproteinase-2; MMP-9: matrix metalloproteinase-9; PDGF: platelet-derived growth factor; A10 cells: vascular smooth muscle cells.

**Table 11:** Antiatherosclerosis of salvianolic acid B in vitro.

Cells/tissues	Effects	Reference
VSMCs	Nrf2-ARE signaling pathway, activation of NQO1.	Hur et al., 2010 [71]

H-monDC	Supress activation of PPAR*γ* nuclear translocation, expression of CD40, CD86, CD1a, and HLA-DR, expression of IL-12 and IL-10, production of TNF-*α*, regulation of TLR4, myeloid differentiation of primary reactive protein 88, p38-MAPK signaling pathway.	Sun et al., 2011 [72]

Macrophage	Reduce mLDL uptake, antagonize CD36.	Bao et al., 2012 [73]

HASMCs	MMPs protein synthesis, downregulate ERK1/2, downregulate c-JNK.	Lin et al., 2007 [74]

Note: H-monDC: human monocyte-derived dendritic cells; Nrf2: nuclear factor 2-related factor 2; NQO1: NAD(P)H quinine oxidoreductase-1; PARP: poly ADP-ribose polymerase; ARE: antioxidant responsive element; VSMC: vascular smooth muscle cells; IL: interleukin; TLR: toll like receptor; TNF: tumor necrosis factor.

**Table 12:** Inhibiting left ventricular remodeling of salvianolic acid B in vivo and in vitro.

Type	Cells/tissues	Effects	Reference
In vitro research	LX-2	p38 signaling pathway, cross effect from the Smad to ERK signaling pathway, synthesis of type I collagen,	Lv and Xu, 2012 [76]
	HL-60	MMP-9 CD, cardiac fibroblast migration, collagen synthesis, secretion of cytokine (ICAM, TNF-*α*, IL-6, and sVCAM-1), expression of VEGF.	Jiang et al., 2010 [77]
	PC12	A-beta aggregation, fibril formation, cellular toxicity of aged A-beta.	Tang and Zhang, 2011 [78]

Type	Organ/animals	Effects	Reference

In vivo research	Rat heart	Activity of MMP-9, increase the thickness of the left ventricular wall, improve the contraction of the heart, reduce cardiac fibrosis.	Wang et al., 2011 [75]

Note: MMP-9 CD: catalytic domain of MMP-9; LX-2: stellate cell lines; VCAM-1: vascular cell adhesion molecule-1; ICAM-1: intercellular adhesion molecule 1; IL: interleukin; TNF: tumor necrosis factor; MMP-9 CD: catalytic domain of MMP-9; ERK: extracellular-signal-regulated kinase; VEGF: vascular endothelial growth factor receptor.

**Table 13:** Anti-arrhythmic of salvianolic acid B in vitro.

Cells/tissues	Effects	Reference
Rat ventricular myocytes	*I*_NA_ kinetics inactivation, prolong APD, then affect EAD, increase APD.	Yoon et al., 2004 [79]

Note: APD: action potential duration; EAD: early after depolarization.

## Abbreviations

8-OHDG: 8-Hydroxy-2-deoxyguanosine
A10 cells: Vascular smooth muscle cells
ABC: Acclerating blood circulation
AIF: Apoptosis inducing factor
APD: Action potential duration
ARE: Antioxidant responsive element
ATF4: Activating transcription factor 4
BA: Basilar artery
BK_Ca_: Iberiotoxin-sensitive Ca^2+^-activated K^+^ current
BSS: Blood stasis syndrome
CPK: Creatine phosphokinase
CVDs: Cardiovascular diseases
DLA: 3,4-Dihydroxy-phenyl lactic acid
dmLSB: Dimethyl lithospermate B
DPPH: 1,1-Diphenyl-2-picryl-hydrazyl
DSP: Danshen dripping pill
EAD: Early after depolarization
ECV304: Human umbilical vein endothelial cells
EGb 761: Extract ginkgo biloba 761
eIF2a: Eukaryotic translation initiation factor 2a
eNOS: Endothelial nitric oxide synthase
ERK: Extracellular-signal-regulated kinase
ET-1: Endothelin-1
GRP78: Glucose-regulated protein 78
H_2_O_2_: Hydrogen peroxide
HAEC: Human aortic endothelial cells
HASMCs: Human aortic smooth muscle cells
HEK293T cell: Human embryonic kidney cells
HG: High glucose
H-monDC: Human monocyte-derived dendritic cells
HO: The heme oxigenase
HUVECs: Human umbilical vein endothelial cells
I/R: Ischemia and reperfusion
*I*_Ca,L_: L-type Ca^2+^ current
ICAM-1: Intercellular adhesion molecule 1
*I*_K_: K^+^ Current
IL: Interleukin
IL-1b: Interleukin-1b
*I*_Na_: Na^+^ current
iNOS: Induced nitric oxide synthase
JAK: Januskinase
JNK: c-Jun N-terminal kinase
L-arginine: Left-handed arginine
LDH: Lactate dehydrogenase
LDQ: Lipophilic diterpenoid quinines
LPS: Lipopolysaccharide
LV: Left ventricular
LX-2: Stellate cell lines
MDA: Malondialdehyde
MI: Myocardial infarction
MLB: Magnesium lithospermate B
MMP-2: Matrix metalloproteinase-2
MMP-9 CD: Catalytic domain of MMP-9
MMP-9: Matrix metalloproteinase-9
NADPH: Nicotinamide Adenine Dinucleotide Phosphate
NF-*κ*B: Nuclear factor *κ*B
NO: Nitric oxide
NQO1: NAD(P)H quinine oxidoreductase-1
Nrf2: Nuclear factor 2-related factor 2
O^2−^: Superoxide anion radicals
OLETF: Otsuka Long-Evans Tokushima Fatty
ox-LDL: Oxidation of low-density lipoprotein
p38 MAPK: p38 mitogen-activated protein kinase
PAI-1: Plasminogen activator inhibitor type 1
PARP: Poly(ADP-ribose) polymerase
PBCRBS: Promoting blood circulation and removing blood stasis
PDGF: Platelet-derived growth factor
PERK: Pancreatic ER kinase (PKR)-like ER kinase
PI3K: Phosphatidylinositol-13-kinase
PLB: Phospholamban
PMA: Phorbol-12-myristate-13-acetate
PR: Photochemical reaction
rCMEC: Rat cerebral microvascular endothe-lial cells
RCTs: Randomized controlled trials
ROS: Reactive oxygen species
SAB: Salvianolic acid B
SAH: Subarachnoid hemorrhage
Sal B: Salvianolic acid B
SAPK: Stress-activatedproteinkinase
SCs: Schwann cells
SERCA2a: Sarco/endoplasmic reticulum ATPase 2a
SIPA: Shear-induced platelet aggregation
SM: Salvia miltiorrhiza
SME: Salvia hydroalcoholic extract
SOD: Superoxide dismutase
SRs: Systematic reviews
TAB1: TGF-*β*-activated protein kinase 1 binding protein 1
TBI: Traumatic brain injury
TLR: Toll like receptor
TM: Thrombomodulin
TMP: Tetramethylpyrazine
TNF: Tumor necrosis factor
t-PA: Tissue-type plasminogen activator
TsI: Tanshinone I
TsIIA: Tanshinone IIA
TsIIB: Tanshinone IIB
TUNEL: Terminal deoxynucleotidyl transferase-mediated dUTP nick end labeling
VCAM-1: Vascular cell adhesion molecule-1
VEGF: Vascular endothelial growth factor receptor
VSMC: Vascular smooth muscle cells
WSC: Water-soluble compounds.

## References

[1] Zhou L, Zuo Z, Chow MSS (2005). Danshen: an overview of its chemistry, pharmacology, pharmacokinetics, and clinical use. *Journal of Clinical Pharmacology*.

[2] Wu B, Liu M, Zhang S (2007). Dan shen agents for acute ischaemic stroke. *Cochrane Database of Systematic Reviews*.

[3] Wang C, Zhao X, Mao S, Wang Y, Cui X, Pu Y (2006). Management of SAH with traditional Chinese medicine in China. *Neurological Research*.

[4] Oda M, Yokomori H, Han JY (2006). Regulatory mechanisms of hepatic microcirculatory hemodynamics: hepatic arterial system. *Clinical Hemorheology and Microcirculation*.

[5] Horie Y, Han JY, Mori S (2005). Herbal cardiotonic pills prevent gut ischemia/reperfusion-induced hepatic microvascular dysfunction in rats fed ethanol chronically. *World Journal of Gastroenterology*.

[6] Xing HC, Li LJ, Xu KJ (2005). Effects of Salvia miltiorrhiza on intestinal microflora in rats with ischemia/reperfusion liver injury. *Hepatobiliary and Pancreatic Diseases International*.

[7] Chen CG, Wang YP (2006). Magnesium lithospermate B ameliorates renal cortical microperfusion in rats. *Acta Pharmacologica Sinica*.

[8] Hoffmann SC, Kampen RL, Amur S (2002). Molecular and immunohistochemical characterization of the onset and resolution of human renal allograft ischemia-reperfusion injury. *Transplantation*.

[9] Bando Y, Tsukamoto Y, Katayama T (2004). ORP150/HSP12A protects renal tubular epithelium from ischemia-induced cell death. *FASEB Journal*.

[10] Chen Y, Ruan Y, Li L (2003). Effects of Salvia miltiorrhiza extracts on rat hypoxic pulmonary hypertension, heme oxygenase-1 and nitric oxide synthase. *Chinese Medical Journal*.

[11] Reignier J, Sellak H, Lemoine R (1997). Prevention of ischemia-reperfusion lung injury by sulfated Lewisa pentasaccharide. *Journal of Applied Physiology*.

[12] Feldman LJ, Himbert D, Juliard JM (2000). Reperfusion syndrome: relationship of coronary blood flow reserve to left ventricular function and infarct size. *Journal of the American College of Cardiology*.

[13] Hu P, Luo GA, Zhao ZZ, Jiang ZH (2005). Quantitative determination of four diterpenoids in radix *Salviae miltiorrhizae* using LC-MS-MS. *Chemical and Pharmaceutical Bulletin*.

[14] Han JY, Fan JY, Horie Y (2008). Ameliorating effects of compounds derived from *Salvia miltiorrhiza* root extract on microcirculatory disturbance and target organ injury by ischemia and reperfusion. *Pharmacology and Therapeutics*.

[15] Ho JHC, Hong CY (2011). Salvianolic acids: small compounds with multiple mechanisms for cardiovascular protection. *Journal of Biomedical Science*.

[16] Jia Y, Huang F, Zhang S, Leung SW (2012). Is danshen (*Salvia miltiorrhiza*) dripping pill more effective than isosorbide dinitrate in treating angina pectoris? A systematic review of randomized controlled trials. *International Journal of Cardiology*.

[17] Shi ZX, Li G (2009). Comparative analysis on the major constituents in radix *Salvia miltiorrhizae* injectable preparations. *China Pharmacy*.

[18] Luo WB, Dong L, Wang YP (2002). Effect of magnesium lithospermate B on calcium and nitric oxide in endothelial cells upon hypoxia/reoxygenation. *Acta Pharmacologica Sinica*.

[19] Sun LQ, Zhao J, Zhang TT (2012). Protective effects of salvianolic acid B on schwann cells apoptosis induced by high glucose. *Neurochemical Research*.

[20] Zhang HS, Wang SQ (2006). Salvianolic acid B from Salvia miltiorrhiza inhibits tumor necrosis factor-*α* (TNF-*α*)-induced MMP-2 upregulation in human aortic smooth muscle cells via suppression of NAD(P)H oxidase-derived reactive oxygen species. *Journal of Molecular and Cellular Cardiology*.

[21] Liu CS, Cheng Y, Hu JF, Zhang W, Chen NH, Zhang JT (2006). Comparison of antioxidant activities between salvianolic acid B and *Ginkgo biloba* extract (EGb 761). *Acta Pharmacologica Sinica*.

[22] Zhao GR, Zhang HM, Ye TX (2008). Characterization of the radical scavenging and antioxidant activities of danshensu and salvianolic acid B. *Food and Chemical Toxicology*.

[23] Ma C, Yao Y, Yue QX (2011). Differential proteomic analysis of platelets suggested possible signal cascades network in platelets treated with salvianolic acid B. *PLoS ONE*.

[24] Mu XY (2009). Influence of salvianolate to platelet aggregation and MMP-9 of patients with unstable angina. *Chinese Medical Herald*.

[25] Radomski MW, Palmer RMJ, Moncade S (1990). An L-arginie nitric oxide pathway present in human platelets regulates aggregation. *Proceedings of the National Academy of Sciences of the United States of America*.

[26] Radomski MW, Palmer RMJ, Moncada S (1990). Characterization of the L-arginine: nitric oxide pathway in human platelets. *British Journal of Pharmacology*.

[27] Freedman JE, Loscalzo J, Barnard MR, Alpert C, Keaney JF, Michelson AD (1997). Nitric oxide released from activated platelets inhibits platelet recruitment. *The Journal of Clinical Investigation*.

[28] Li M, Zhao C, Wong RNS, Goto S, Wang Z, Liao F (2004). Inhibition of shear-induced platelet aggregation in rat by tetramethylpyrazine and salvianolic acid B. *Clinical Hemorheology and Microcirculation*.

[29] Wu YP, Zhao XM, Pan SD (2008). Salvianolic Acid B inhibits platelet adhesion under conditions of flow by a mechanism involving the collagen receptor *α*2*β*1. *Thrombosis Research*.

[30] Lay IS, Chiu JH, Shiao MS, Lui WY, Wu CW (2003). Crude extract of Salvia miltiorrhiza and salvianolic acid B enhance in vitro angiogenesis in murine SVR endothelial cell line. *Planta Medica*.

[31] Lee BW, Chun SW, Kim SH (2011). Lithospermic acid B protects beta-cells from cytokine-induced apoptosis by alleviating apoptotic pathways and activating anti-apoptotic pathways of Nrf2-HO-1 and Sirt1. *Toxicology and Applied Pharmacology*.

[32] Blanc A, Pandey NR, Srivastava AK (2003). Synchronous activation of ERK 1/2, p38mapk and PKB/Akt signaling by H_2_O_2_ in vascular smooth muscle cells: potential involvement in vascular disease. *International journal of molecular medicine*.

[33] Liu CL, Xie LX, Li M, Durairajan SSK, Goto S, Huang JD (2007). Salvianolic acid B inhibits hydrogen peroxide-induced endothelial cell apoptosis through regulating PI3K/Akt signaling. *PLoS ONE*.

[34] Du CS, Yang RF, Song SW, Wang YP (2010). Magnesium lithospermate B protects cardiomyocytes from ischemic injury via inhibition of TAB1-p38 apoptosis signaling. *Frontiers in Pharmacology*.

[35] Xu LL, Deng YP, Feng LX (2011). Cardio protection of salvianolic acid B through inhibition of apoptosis network. *PLoS ONE*.

[36] Han X, Liu JX, Li XZ (2011). Salvianolic acid B inhibits autophagy and protects starving cardiac myocytes. *Acta Pharmacologica Sinica*.

[37] Tang M, Zhang J (2002). Prostate apoptosis response-4 involved in the protective effect of salvianolic acid B against amyloid *β* peptide-induced damage in PC12 cells. *Japanese Journal of Pharmacology*.

[38] Liu X, Chen R, Shang Y, Jiao B, Huang C (2009). Superoxide radicals scavenging and xanthine oxidase inhibitory activity of magnesium lithospermate B from *Salvia miltiorrhiza*. *Journal of Enzyme Inhibition and Medicinal Chemistry*.

[39] Hung HH, Chen YL, Lin SJ (2001). A salvianolic acid B-rich fraction of *Salvia miltiorrhiza* induces neointimal cell apoptosis in rabbit angioplasty model. *Histology and Histopathology*.

[40] Yang J, Zhang G, Tian J (2010). Cardioprotective effect of SMND-309, a novel derivate of salvianolic acid B on acute myocardial infarction in rats. *Basic and Clinical Pharmacology and Toxicology*.

[41] Wang W, Hu GY, Wang YP (2006). Selective modulation of L-type calcium current by magnesium lithospermate B in guinea-pig ventricular myocytes. *Life Sciences*.

[42] Pan C, Lou L, Huo Y (2011). Salvianolic acid B and Tanshinone IIA attenuate myocardial ischemia injury in mice by no production through multiple pathways. *Therapeutic Advances in Cardiovascular Disease*.

[43] Wang J, Zhang Y, Guo LL (2011). Salvianolic acid B inhibits the TLR4-NF*κ*B-TNF*α* pathway and attenuates neonatal rat cardiomyocyte injury induced by lipopolysaccharide. *Chinese Journal of Integrative Medicine*.

[44] He H, Shi M, Zeng X (2008). Cardioprotective effect of salvianolic acid B on large myocardial infarction mediated by reversing upregulation of leptin, endothelin pathways, and abnormal expression of SERCA2a, phospholamban in rats. *Journal of Ethnopharmacology*.

[45] Yang LM, Xiao YL, Ou-Yang JH (2003). Inhibition of magnesium lithospermate B on the c-Jun N-terminal kinase 3 mRNA expression in cardiomyocytes encountered ischemia/reperfusion injury. *Acta Pharmaceutica Sinica*.

[46] Chang CZ, Wu SC, Kwan AL (2011). Magnesium lithospermate B alleviates the production of endothelin-1 through an NO-dependent mechanism and reduces experimental vasospasm in rats. *Acta Neurochirurgica*.

[47] Chen YH, Lin SJ, Ku HH (2001). Salvianolic acid B attenuates VCAM-1 and ICAM-1 expression in TNF-*α*-treated human aortic endothelial cells. *Journal of Cellular Biochemistry*.

[48] Kim SH, Kim SH, Choi M (2010). Natural therapeutic magnesium lithospermate B potently protects the endothelium from hyperglycaemia-induced dysfunction. *Cardiovascular Research*.

[49] Zhou Z, Liu Y, Miao AD, Wang SQ (2005). Salvianolic acid B attenuates plasminogen activator inhibitor type 1 production in TNF-*α* treated human umbilical vein endothelial cells. *Journal of Cellular Biochemistry*.

[50] Wu HL, Li YH, Lin YH (2009). Salvianolic acid B protects human endothelial cells from oxidative stress damage: a possible protective role of glucose-regulated protein 78 induction. *Cardiovascular Research*.

[51] Yang Q, Wang S, Xie Y (2010). Effect of Salvianolic acid b and paeonol on blood lipid metabolism and hemorrheology in myocardial schemia rabbits induced by pituitruin. *International Journal of Molecular Sciences*.

[52] Shi CS, Huang HC, Wu HL (2007). Salvianolic acid B modulates hemostasis properties of human umbilical vein endothelial cells. *Thrombosis Research*.

[53] Wang F, Liu YY, Liu LY (2009). The attenuation effect of 3,4-dihydroxy-phenyl lactic acid and salvianolic acid B on venular thrombosis induced in rat mesentery by photochemical reaction. *Clinical Hemorheology and Microcirculation*.

[54] Zhang HF, Chen XQ, Hu GY, Wang YP (2010). Magnesium lithospermate B dilates mesenteric arteries by activating BK Ca currents and contracts arteries by inhibiting KV currents. *Acta Pharmacologica Sinica*.

[55] Lam FFY, Seto SW, Kwan YW, Yeung JHK, Chan P (2006). Activation of the iberiotoxin-sensitive BKCa channels by salvianolic acid B of the porcine coronary artery smooth muscle cells. *European Journal of Pharmacology*.

[56] Chen YC, Jinn TR, Chung TY, Li FY, Fan RJ, Tc Tzen J (2010). Magnesium lithospermate B extracted from Salvia miltiorrhiza elevats intracellular Ca^2+^ level in SH-SY5Y cells. *Acta Pharmacologica Sinica*.

[57] Wang W, Hu GY, Wang YP (2006). Selective modulation of L-type calcium current by magnesium lithospermate B in guinea-pig ventricular myocytes. *Life Sciences*.

[58] Lam FFY, Yeung JHK, Kwan YW, Chan KM, Or PMY (2006). Salvianolic acid B, an aqueous component of danshen (*Salvia miltiorrhiza*), relaxes rat coronary artery by inhibition of calcium channels. *European Journal of Pharmacology*.

[59] Jang SI, Kim HJ, Kim YJ, Jeong SI, You YO (2006). Tanshinone IIA inhibits LPS-induced NF-*κ*B activation in RAW 264.7 cells: possible involvement of the NIK-IKK, ERK1/2, p38 and JNK pathways. *European Journal of Pharmacology*.

[60] Jang SI, Jeong SI, Kim KJ (2003). Tanshinone IIA from salvia miltiorrhiza inhibits inducible nitric oxide synthase expression and production of TNF-*α*, IL-1*β* and IL-6 in activated RAW 264.7 cells. *Planta Medica*.

[61] Sun AJ, Liu HY, Wang SJ (2011). Salvianolic acid B suppresses maturation of human monocyte-derived dendritic cells by activating PPAR*γ*. *British Journal of Pharmacology*.

[62] Chung CS, Lin YL, Huang B (2011). Salvianolic acid B suppresses IFN-*γ*-induced JAK/STAT1 activation in endothelial cells. *Thrombosis Research*.

[63] Wang SX, Hu LM, Gao XM, Guo H, Fan GW (2010). Anti-inflammatory activity of salvianolic acid B in microglia contributes to its neuroprotective effect. *Neurochemical Research*.

[64] Cheng CC, Yang SP, Lin WS (2012). Magnesium lithospermate B mediates anti-inflammation targeting activator protein-1 and nuclear factor-*κ* B signaling pathways in human peripheral T lymphocytes. *International Immunopharmacology*.

[65] Chen T, Liu W, Chao X (2011). Salvianolic acid B attenuates brain damage and inflammation after traumatic brain injury in mice. *Brain Research Bulletin*.

[66] Chen YL, Hu CS, Lin FY (2006). Salvianolic acid B attenuates cyclooxygenase-2 expression in vitro in LPS-treated human aortic smooth muscle cells and in vivo in the apolipoprotein-E-deficient mouse aorta. *Journal of Cellular Biochemistry*.

[67] Xie LX, Durairajan SSK, Lu JH (2010). The effect of salvianolic acid B combined with laminar shear stress on TNF-*α*-stimulated adhesion molecule expression in human aortic endothelial cells. *Clinical Hemorheology and Microcirculation*.

[68] Hur KY, Seo HJ, Kang ES (2008). Therapeutic effect of magnesium lithospermate B on neointimal formation after balloon-induced vascular injury. *European Journal of Pharmacology*.

[69] Pan CH, Chen CW, Ming-Jyh S (2012). Salvianolic acid B inhibits SDF-1*α*-stimulated cell proliferation and migration of vascular smooth muscle cells by suppressing CXCR4 receptor. *Vascular Pharmacology*.

[70] Luo P, Tan Z, Zhang Z, Li H, Mo Z (2008). Inhibitory effects of salvianolic acid B on the high glucose-induced mesangial proliferation via NF-*κ*B-dependent pathway. *Biological and Pharmaceutical Bulletin*.

[71] Hur KY, Kim SH, Choi MA (2010). Protective effects of magnesium lithospermate B against diabetic atherosclerosis via Nrf2-ARE-NQO1 transcriptional pathway. *Atherosclerosis*.

[72] Sun AJ, Liu HY, Wang SJ (2011). Salvianolic acid B suppresses maturation of human monocyte-derived dendritic cells by activating PPAR*γ*. *British Journal of Pharmacology*.

[73] Bao Y, Wang L, Xu YN (2012). Salvianolic acid B inhibits macrophage uptake of modified low density lipoprotein (mLDL) in a scavenger receptor CD36-dependent manner. *Atherosclerosis*.

[74] Lin SJ, Lee IT, Chen YH (2007). Salvianolic acid B attenuates MMP-2 and MMP-9 expression in vivo in apolipoprotein-E-deficient mouse aorta and in vitro in LPS-treated human aortic smooth muscle cells. *Journal of Cellular Biochemistry*.

[75] Wang YH, Xu F, Chen J (2011). Matrix metalloproteinase-9 induces cardiac fibroblast migration, collagen and cytokine secretion: inhibition by salvianolic acid B from *Salvia miltiorrhiza*. *Phytomedicine*.

[76] Lv Z, Xu L (2012). Salvianolic Acid B inhibits ERK and p38 MAPK signaling in TGF-*β*1-stimulated human hepatic stellate cell Line (LX-2) via distinct pathways. *Evidence-Based Complementary and Alternative Medicine*.

[77] Jiang B, Chen J, Xu L (2010). Salvianolic acid B functioned as a competitive inhibitor of matrix metalloproteinase-9 and efficiently prevented cardiac remodeling. *BMC Pharmacology*.

[78] Tang MK, Zhang JT (2001). Salvianolic acid B inhibits fibril formation and neurotoxicity of amyloid beta-protein in vitro. *Acta Pharmacologica Sinica*.

[79] Yoon JY, Ahn SH, Oh H (2004). A novel Na^+^ channel agonist, dimethyl lithospermate B, slows Na^+^ current inactivation and increases action potential duration in isolated rat ventricular myocytes. *British Journal of Pharmacology*.

[80] Xu H, Chen KJ (2010). Making evidence-based decisions in the clinical practice of integrative medicine. *Chinese Journal of Integrative Medicine*.

[81] Chen KJ (2008). Clinical service of Chinese medicine. *Chinese Journal of Integrative Medicine*.

[82] Wang J, Xiong XJ (2012). Current situation and perspectives of clinical study in integrative medicine in China. *Evidence-Based Complementary and Alternative Medicine*.

[83] Wang J, Xiong XJ (2012). Control strategy on hypertension in Chinese medicine. *Evidence-Based Complementary and Alternative Medicine*.

[84] Wang J, Xiong XJ (2012). Outcome measures of Chinese herbal medicine for hypertension: an overview of systematic reviews. *Evidence-Based Complementary and Alternative Medicine*.

[85] Liu MY, Chen KJ (2012). Convergence: the tradition and the modern. *Chinese Journal of Integrative Medicine*.

[86] Wang J, Wang PQ, Xiong XJ (2012). Current situation and re-understanding of syndrome and formula syndrome in Chinese medicine. *Internal Medicine*.

[87] Robinson N (2011). Integrative medicine—traditional Chinese medicine, a model?. *Chinese Journal of Integrative Medicine*.

[88] Liu Y, Yin HJ, Shi DZ, Chen KJ (2012). Chinese herb and formulas for promoting blood circulation and removing blood stasis and antiplatelet therapies. *Evidence-Based Complementary and Alternative Medicine*.

[89] Xu H, Chen KJ (2011). Integrating traditional medicine with biomedicine towards a patient-centered healthcare system. *Chinese Journal of Integrative Medicine*.

[90] Chen KJ, Shi DZ, Xu H (2006). XS0601 reduces the incidence of restenosis: a prospective study of 335 patients undergoing percutaneous coronary intervention in China. *Chinese Medical Journal*.

[91] Xu H, Chen K (2008). Integrative medicine: the experience from China. *Journal of Alternative and Complementary Medicine*.

[92] Chen KJ (2010). Where are we going?. *Chinese Journal of Integrative Medicine*.

[93] Xiong XJ, Chu FY, Li HX, He QY (2011). Clinical application of the TCM classic formulae for treating chronic bronchitis. *Journal of Traditional Chinese Medicine*.

[94] Xu H, Chen KJ (2012). Complementary and alternative medicine: is it possible to be mainstream. *Chinese Journal of Integrative Medicine*.

[95] Keji C, Hao X (2003). The integration of traditional Chinese medicine and western medicine. *European Review*.

[96] Xiong XJ, Chu FY, Li HX, He QY (2011). Clinical application of the TCM classic formulae for treating chronic bronchitis. *Journal of Traditional Chinese Medicine*.

[97] Xiong XJ, Wang J (2010). Discussion of related problems in herbal prescription science based on objective indications of herbs. *Journal of Chinese Integrative Medicine*.

[98] Xiong XJ, Wang J (2011). Experience of diagnosis and treatment of exogenous high-grade fever. *Journal of Chinese Integrative Medicine*.

[99] Wang J, Xiong XJ (2011). Explaining syndromes of decoction for removing blood stasis in chest. *Zhongguo Zhong Yao Za Zhi*.

